# Interpretation of concurrent positive skin tests to prophylactic antibiotics and rocuronium

**DOI:** 10.1186/s40981-021-00468-2

**Published:** 2021-08-23

**Authors:** Kazuhiro Nagumo, Tomonori Takazawa, Shigeru Saito

**Affiliations:** 1 Department of Anesthesiology, Gunma University Graduate School of Medicine, Gunma, Japan; 2 Intensive Care Unit, Gunma University Hospital, 3-39-15 Showa-machi, Maebashi, Gunma, 371-8511 Japan

To the Editor,

We read with great interest the article by Yasuda et al. on concurrent positive skin tests to prophylactic antibiotics and rocuronium in two patients [1]. The authors hypothesized the possible synergistic effect of antibiotics and neuromuscular blocking agents (NMBAs) on anaphylactic reactions. As evidence to support their hypothesis, they quoted a previous study that showed that patients with positive skin tests to antibiotics were more likely to have positive skin tests to NMBAs [2]. However, we believe that the evidence to prove their hypothesis is insufficient for the reasons described below.

First, since the authors did not indicate the concentration of drugs being tested when the skin test was positive, the possibility of false-positive skin test results cannot be ruled out. In general, false positives should be kept in mind when skin tests are positive, especially when they are positive for two or more drugs. NMBAs, in particular, are well known as drugs that are prone to producing false-positive results. Adherence to the maximum concentrations of drugs recommended in the guidelines is crucial to avoid false-positive results in skin testing [3]. Further, lack of information on both the method of determining the positive skin test and photographs of the skin test results is another reason why we suspect there could have been false-positive results in these two cases.

Next, it is possible that case 2 might not even have developed anaphylaxis, since other possible causes can explain the observed decrease in blood pressure and percutaneous oxygen saturation (SpO_2_): hypotension might have occurred secondary to the effect of epidural anesthesia and anesthesia-inducing drugs. Since a decrease in SpO_2_ occurs after pneumoperitoneum, the cause for decrease in SpO_2_ could likely have been atelectasis due to elevation of the diaphragm. Additionally, no increase in serum tryptase was observed. Applying the clinical score of perioperative anaphylaxis to case 2 gives a total of 6 points: 6 points for severe hypotension, 2 points for a poor response to the standard dose of sympathomimetics, 3 points for the onset of cardiovascular features within 15 min of a possible intravenous trigger (including our estimates), − 1 point for neuraxial regional anesthesia, and − 4 points for absence of tryptase elevation [4]. A score of less than 8 is defined as “unlikely to be an immediate hypersensitivity reaction”, suggesting that case 2 was not anaphylaxis [4]. Further, the cut-off value for serum tryptase mentioned by the authors (5.7 ng/ml) is different from the cut-off value that is generally used (11.4 ng/ml). Alternatively, basal tryptase × 1.2 + 2 ng/ml is the recommended gold standard threshold in evaluating mast cell activation [5].

In summary, the authors’ hypothesis is promising and worth investigating. However, their claim that both cases represented anaphylaxis cases with positive skin tests to muscle relaxants and antibiotics might not be correct for the reasons given above. We recommend the combined use of in vitro tests with high specificity, for example, the basophil activation test, when skin tests show ambiguous results, including positivity for multiple drugs.

## Data Availability

Not applicable.

## Abbreviations

NMBA: Neuromuscular blocking agent
SpO_2_: Percutaneous oxygen saturation
